# Ad hoc efforts for advancing data science education

**DOI:** 10.1371/journal.pcbi.1007695

**Published:** 2020-05-07

**Authors:** Orianna DeMasi, Alexandra Paxton, Kevin Koy

**Affiliations:** 1 Department of Computer Science, University of California, Davis, California, United States of America; 2 Department of Psychological Sciences, University of Connecticut, Storrs, Connecticut, United States of America; 3 Center for the Ecological Study of Perception and Action, University of Connecticut, Storrs, Connecticut, United States of America; 4 IDEO, San Francisco, California, United States of America; University of Toronto, CANADA

## Abstract

With increasing demand for training in data science, extracurricular or “ad hoc” education efforts have emerged to help individuals acquire relevant skills and expertise. Although extracurricular efforts already exist for many computationally intensive disciplines, their support of data science education has significantly helped in coping with the speed of innovation in data science practice and formal curricula. While the proliferation of ad hoc efforts is an indication of their popularity, less has been documented about the needs that they are designed to meet, the limitations that they face, and practical suggestions for holding successful efforts. To holistically understand the role of different ad hoc formats for data science, we surveyed organizers of ad hoc data science education efforts to understand how organizers perceived the events to have gone—including areas of strength and areas requiring growth. We also gathered recommendations from these past events for future organizers. Our results suggest that the perceived benefits of ad hoc efforts go beyond developing technical skills and may provide continued benefit in conjunction with formal curricula, which warrants further investigation. As increasing numbers of researchers from computational fields with a history of complex data become involved with ad hoc efforts to share their skills, the lessons learned that we extract from the surveys will provide concrete suggestions for the practitioner-leaders interested in creating, improving, and sustaining future efforts.

## Author summary

Large datasets are becoming integral to society broadly and to biological sciences in particular. As a result, demand for sophisticated data skills and experience has skyrocketed and left some individuals scrambling to cross-train and acquire more computational skills. While universities are racing to develop formal curricula to meet this demand, diverse informal efforts have emerged to fill the immediate demand for skills and experience. These “ad hoc” efforts have been playing a vital role in data science education, especially for domain scientists. While some studies have shown specific ad hoc formats to have considerable impact, few studies have focused on these efforts holistically. Here, we survey effort organizers from leading data science institutes and collect lessons learned. We find that efforts are commonly reported to successfully provide opportunities in difficult areas where curricula could improve, such as providing approachable introductions to new skills, increasing diversity of backgrounds, and fostering heterogeneous communities. However, efforts also report challenges fulfilling diverse needs and offer suggestions. In total, the lessons that we collect from these efforts are useful to improve future ad hoc efforts and to inform formal programs, which may be looking for inspiration to design innovative educational formats.

## Introduction

Interest in data science and related fields has surged over the last several years [1]. Typically seen as applying programming ability and statistical knowledge to answer questions derived from domain-specific expertise, data scientists have come into high demand as datasets have grown in size and complexity [2]. While many university curricula are acknowledging the need for data science training and other computationally minded educational opportunities [3–12], formal program structures and course offerings that embrace these new data and techniques can be slow to change.

To bridge the immediate gap between current curricula and the new demands of data science, a tapestry of extracurricular educational opportunities (i.e., opportunities that do not offer any course credit and are not required to complete a degree program) has emerged to provide students with essential data science skills. These ad hoc education efforts can take a variety of formats—including hours-long workshops, week-long boot camps, and semester-long research projects—and are intended to complement existing formal educational structures [13–15] by embracing new tools and pedagogy as they emerge [16, 17]. These efforts are spearheaded by practitioner-leaders—data scientists across career stages and paths who may or may not have formal teaching expertise but want to share their knowledge with others. Researchers from fields with a strong tradition of complex data and computational skills—like computational biology—have been some of the fastest to jump into these educational opportunities, eager to share their skills with burgeoning data scientists and established researchers integrating data science into fields in the “long tail” of big data and computational work.

Previous studies have considered the benefit of specific ad hoc formats like hack weeks [13], summer programs [14], and workshops [8, 15, 18–20]. Some of this work has indicated that—along with filling educational gaps temporarily created by data science’s rapid growth—ad hoc efforts may also help address more systemic weaknesses through innovative paradigms developed across rapid iterations [13]. Other work has addressed the institutional change of data science education [11] and how to design formal efforts or courses related to computational skills [10, 21]. Prior work has also considered lessons learned from individual event formats, such as short courses or workshops [8, 19, 20, 22–24], mentor–mentee relationships [25], and summer programs [26]. However, to our knowledge, no study has yet looked holistically at the benefits that different extracurricular formats can provide and has extracted lessons learned for future efforts and novel formats.

To formally understand the breadth, impacts, and opportunities for growth for ad hoc efforts broadly, we surveyed organizers from a variety of efforts. These efforts were all organized at the Moore-Sloan Data Science Environments (MSDSEs), an early initiative to promote interdisciplinary data science research, education, and communities at New York University (NYU), the University of Washington (UW), and the University of California, Berkeley (UC Berkeley). This survey asked organizers to be constructively self-critical to share lessons learned for future efforts through a balanced view of the efforts with which they had been involved. (For survey details, see “Materials and methods” section.) In addition to describing their efforts, we asked them to outline the goals of their events, to explicitly describe the ways in which they were successful and unsuccessful in relation to those goals, to list ways in which they (or others) would change their effort (or similar efforts), and to provide their lessons and thoughts about the future of ad hoc education in data science.

Using these data, we then turned to the major contribution for the current paper: providing concrete guidance to improve future ad hoc education efforts in data science across effort formats. To achieve this, we asked past organizers to reflect on their experiences and provide suggestions for future organizers in a series of structured closed-form and open-form questions. From the open-ended responses, we used qualitative research methods [27, 28] to extract a codebook for capturing recurring themes. (For more on how the codebook was developed, see “Materials and methods”.) This codebook—a secondary contribution of the current work—is intended to be both a guide to the specific responses for our survey and a tool for future qualitative and quantitative explorations. These lessons learned additionally provide us an opportunity to explore implications for the future of ad hoc data science education—especially within evolving and increasingly rich formal education structures.

## Results

Our survey received 24 total responses, but 2 were excluded because the respondent did not consent to participate in the research. The 22 included responses represented the perspectives of 18 unique organizers on 19 unique efforts (Table 1). The original 24 survey responses represented—to the best of our knowledge—a comprehensive list of ad hoc data science education efforts within the MSDSEs at the time. (There were and are additional ad hoc efforts at each host university, but we restrict our focus to ad hoc education efforts in data science sponsored by an MSDSE.) Therefore, the 22 responses included in these analyses represent a nearly comprehensive list.

**Table 1:** Summary of dataset size, which nearly comprehensively represents ad hoc data science efforts held at the MSDSEs.

	Count
Included responses	22
Unique efforts reviewed	19
Organizers reporting	18

**Abbreviation:** MSDSE, Moore-Sloan Data Science Environment

Many of these efforts represented multiple (e.g., annual) iterations of an event or multiple events in a series, so considerably more events are represented. The data were originally collected as a means to understand how ad hoc efforts in the MSDSE could be improved. Pursuant to UC Berkeley Institutional Review Board (IRB) protocol ID 2017-11-10487, we subsequently obtained consent from the respondents so that the lessons learned could be shared more broadly.

Taken together, the organizers in our survey reported approximately 1,194 participants for the events considered. However, since some organizers noted that the events occurred regularly (e.g., weekly, quarterly), these ad hoc efforts may have included up to 3,554 participants, using the reported frequency and assuming relatively stable rates of participation. While there may have been overlap in participants between events, these ad hoc efforts touched a large number of individuals seeking data science training and experience.

### Types of efforts held at the MSDSEs

The efforts reported in our survey included a variety of formats (see Table 2 for examples). Each of the efforts reported in our survey could generally be characterized along 2 orthogonal axes: high or low investment and long-term or short-term cohesion. Investment captures the amount of resources (e.g., space, funding) and/or efforts required to create the event. Cohesion focuses on the persistence of the effort over time. This does not necessarily mean that the specific individuals involved in the effort will remain the same over time; instead, this captures the persistence of the effort itself.

**Table 2:** Summary of effort types and examples of effort formats. Count indicates the number of survey responses that represented efforts that could be classified as a given type.

Type	Count	Examples
LIST	3	Short-term consulting
HILT	4	Series of mini-workshops; hands-on projects
HIST	11	Focused workshop not on methods or research; hack weeks; concentrated hands-on workshop or speaker series
LILT	4	Recurring presentations on methods; recurring speaker series; consulting on long-term, hands-on projects; long-term research projects

**Abbreviations:** HILT, high investment, long-term cohesion; HIST, high investment, short-term cohesion; LILT, low investment, long-term cohesion; LIST, low investment, short-term cohesion

#### High investment, short-term cohesion

The majority of the MSDSE efforts in our survey were high investment, short-term cohesion (HIST; Table 2), as they required coordination among multiple leaders to create a unified program spanning several days or a week. HIST efforts could include well-known types of ad hoc efforts discussed in other works—for example, hack weeks (i.e., multiday events that mix tutorials and lectures with dedicated time to intensively work on a project [13]) and multiple-day workshops (e.g., Software Carpentry [15]) at all 3 campuses. The majority of the HIST efforts included in our survey were not driven by faculty members, highlighting the openness of ad hoc education effort leadership.

#### Low investment, short-term cohesion

Ad hoc education efforts described as low investment, short-term cohesion (LIST) are often single-day events with much more distributed investment requirements. Examples of LISTs would include other popular formats, such as single-day “un-conferences” [29] focusing on cross-disciplinary analyses of a single type of data or “lightning talks” (i.e., 3- to 10-minute talks) aimed at practically tackling single questions or topics in data science. By their nature, these efforts afford the opportunity for much more targeted events that take advantage of existing strengths within the local community and target specific needs or narrow topics.

#### High investment, long-term cohesion

High investment, long-term cohesion (HILT) efforts require multiple investments (e.g., time, resources, cost) to persist over months or years. To do so, some efforts required hierarchies of training for researcher or software development mentors (e.g., “train the trainer” models). Prototypical HILT efforts reported in our survey included a focus on hands-on research projects or software development through close mentoring relationships for an extended period of time (e.g., semester, summer). While these efforts are rewarding, the required resources present a substantial challenge.

#### Low investment, long-term cohesion

Efforts classified as low investment, long-term cohesion (LILT) exist on longer scales but require relatively little centralized investment. Such efforts are often championed by a single organizer who can set up the structure over a semester or year. For example, LILT efforts could include short consulting sessions, ongoing peer-learning tutorials, and lecture series. The loosely connected structure allows organizers to take advantage of existing community expertise while deepening community ties and broadening community knowledge. These events may build on one another, but their relatively informal structure may impose lower barriers to entry for participants.

### Diverse intended audiences for ad hoc efforts

**Fig 1:**
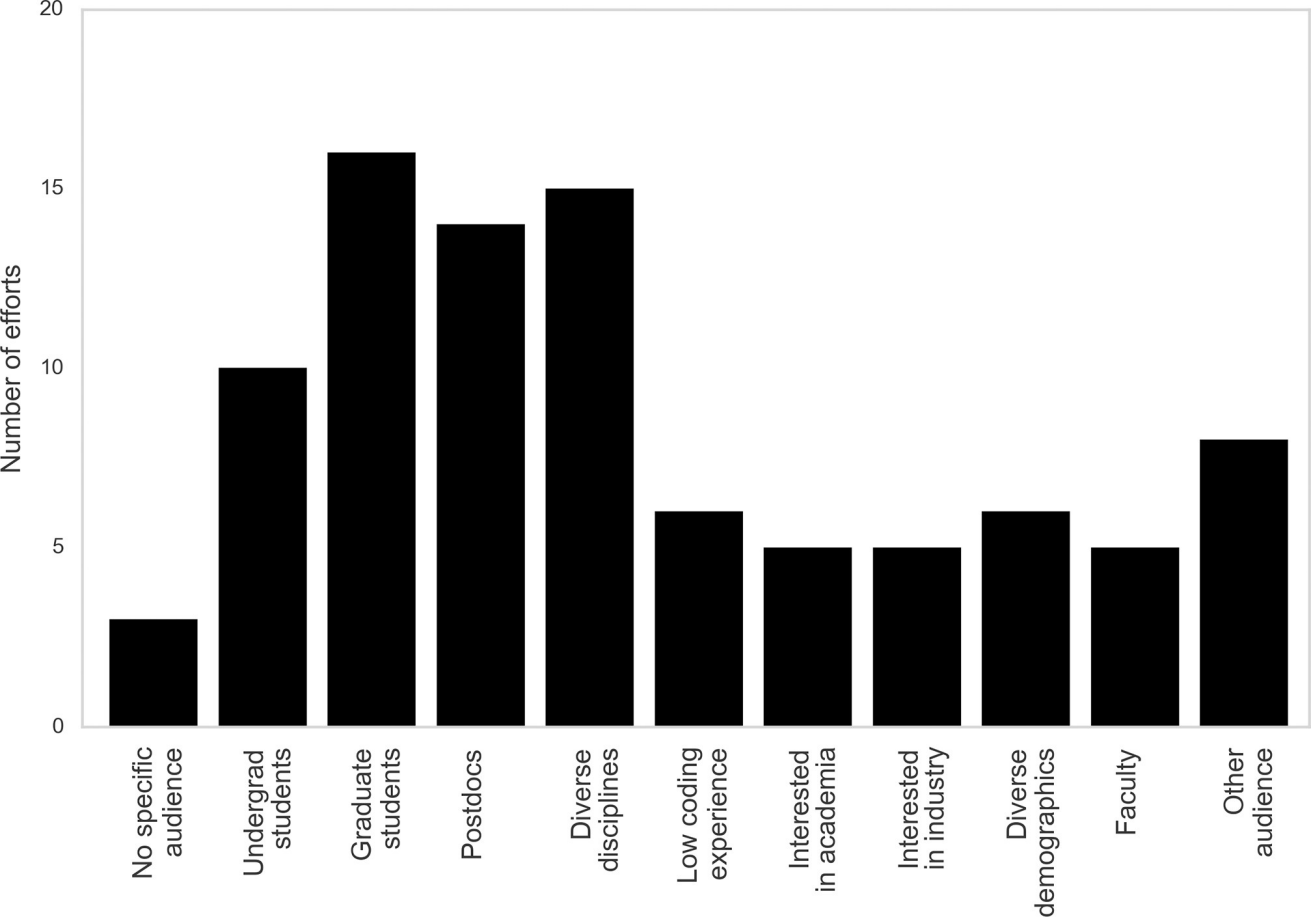
Target audiences reported by efforts. Efforts typically reported more than one target audience, and each audience listed was targeted by multiple efforts. The audiences included in the figure were multiple-choice options for the survey question, except for “Faculty,” which was written into the “Other” option often, as indicated here.

To understand which audiences ad hoc efforts have tried to engage and whether they successfully engaged underserved audiences, we asked organizers to name their target populations using a multiple-answer question on our survey. As seen in Fig 1, each audience listed was targeted by multiple efforts. We tried to be as broad as possible in identifying different kinds of diversity: In addition to using the word “diverse” in reference both to demographics and disciplines, we included a range of other kinds of diversity (including career stage, programming backgrounds, and career goals).

Every respondent indicated targeting more than one of the identified populations, and some indicated additional audiences that were not specified by the survey question. By having various effort structures, as discussed earlier, some ad hoc efforts (especially those with short-term cohesion) can create a lower barrier to entry than formal curricula and thus provide initial contact with data science to diverse audiences. These efforts can also be tuned to meet needs of specific audiences, as they are extracurricular and often relatively brief. By incorporating more diverse audiences, ad hoc efforts can enrich learning outcomes and make data science more accessible.

### Common goals of ad hoc efforts

**Fig 2:**
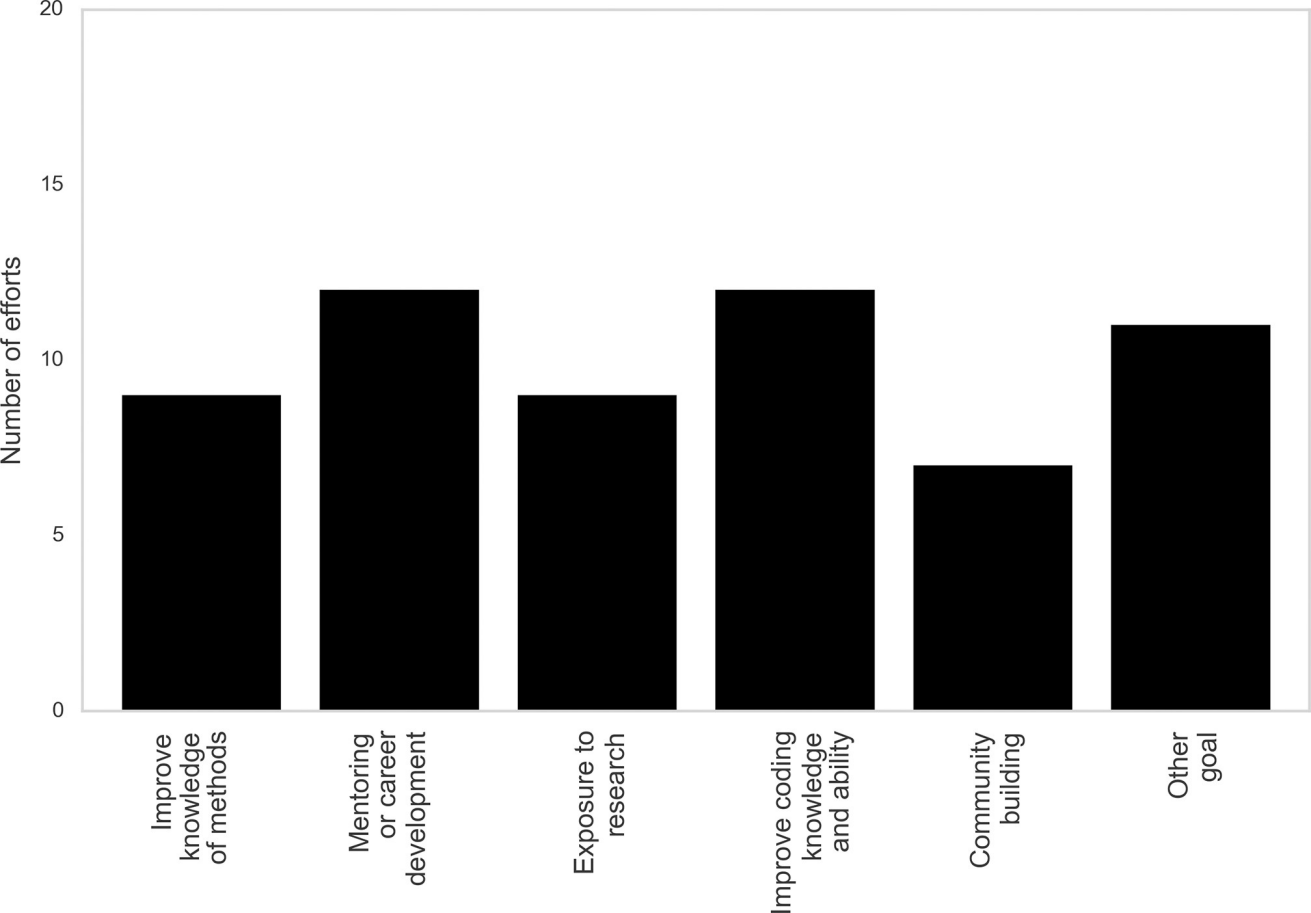
Goals reported by efforts. Community building was not included as a possible multiple-answer response, but it was cited in open-ended responses for approximately a third of all efforts.

Using multiple-answer responses, every respondent indicated that their effort had in mind at least one of 4 listed goals that are not always well met by formal curricula (Fig 2). Many efforts also indicated additional, unlisted goals—with one of the most significant themes being building community and research collaborations. This theme manifested in a variety of ways, but the diverse communities formed at ad hoc events and persisting beyond events were often described as a long-term benefit to research and educational outcomes. By targeting the areas listed, ad hoc education attempts to supplement curricula with novel structures to address traditional challenges or shortcomings of curricula.

### Lessons learned: Things that worked

While many open-ended responses to our question about effort successes were specific to the event or the type of ad hoc effort held, we found 5 general characteristics that commonly emerged as successes of ad hoc education. Most of these characteristics explicitly emerged from a grounded approach (further discussed in the “Materials and methods” section) as codes and can be used to design or plan and evaluate future efforts. Note that the frequency with which these characteristics were reported is likely underestimated due to the current research methods: The open-ended survey questions did not explicitly ask about individual characteristics but instead allowed respondents to volunteer information about whatever stood out to them as successes.

#### Increasing diversity across backgrounds, experiences, perspectives, and skills

By providing approachable introductions on limited time scales, many efforts also reported targeting diversity (e.g., career stages, demographics, disciplines; Fig 1), and 50% of survey responses mentioned successfully engaging a diverse audience. Data science often requires individuals to reach across disciplines. While this diversity sparks exciting research and important discoveries, it can also create barriers both to entry and to progress. By offering small modules with directed foci, ad hoc efforts provide a less daunting environment to data science education that can empower learners and accelerate individuals’ access to new information and skills.

#### Fostering technical skills and research

One of the most common successes across ad hoc efforts seemed to be creating formats that could make a new topic, skill set, and/or technical method approachable. Ad hoc effort organizers frequently mentioned (68.2% of responses) having given participants the opportunity for hands-on experience building and practicing technical skills and research, not just theoretical concepts.

Many of these efforts were specifically designed as introductions to material. University curricula often leave students choosing between taking a formal course or learning the skill on their own. Ad hoc education efforts smoothed the spectrum between these options, helping learners to quickly access new material with expert support. The approachability of tutorial, “hacker” session, and workshop formats is important not only for individuals new to data science broadly but also for those transitioning into new or interdisciplinary areas and awareness of new methods.

Technical skills require significant practice to refine, and ad hoc efforts supported this through structured practice (e.g., direct instruction, project-directed learning) and feedback. As examples, some ad hoc efforts provided supported introductions to new programming libraries with a tutorial format, while others offered opportunities for more practice through a semester-long, hands-on, and mentored research project. However, this success requires further investigation from participants’ perspectives and objective outcomes, as recent work has found null learning effects [30] and thus contradicts work that found positive effort outcomes [13, 19, 20, 31].

#### Fostering nontechnical skills

Similarly, many efforts mentioned providing experience in nontechnical skills (40.9% of responses), such as leading a teaching session at a workshop or mentoring a group of undergraduates through a research project. While nontechnical skills like presenting, mentoring, management, and communication are vital to successful careers in data science, university settings do not always provide supportive environments to build, practice, and refine these skills. Ad hoc education efforts gave opportunities to build novel skill sets commonly seen as outside the scope of standard university curricula.

#### Building enduring communities that improve research

A large proportion of responses indicated that efforts had significant participation (40.9% of responses). Many of these described this participation as building communities around common problems, tools, or experiences and reported that these communities persisted across multiple versions of the effort or beyond the effort. Because efforts can attract diverse audiences, many efforts reported that the newly formed communities included members who otherwise would probably not have connected. In addition to the broad benefits that emerge from being part of a community, organizers also reported specific productive collaborations that stemmed from certain efforts.

### Lessons learned: Things that didn't work

While many organizers who took our survey felt that their effort had been somewhat successful, all but 3 efforts elaborated on room for improvement. The majority of efforts (86.4%) mentioned specific ways that they could refine logistics for their effort or similar kinds of efforts (e.g., scheduling time, organizing materials). However, the organizers’ responses also mentioned more general opportunities for improvement. We grouped the general responses into 4 themes.

As with successes, we note that each of these challenges are likely underreported, due to the open nature of our survey question about effort shortcomings and the lack of participant reports.

#### Unclear expectations of participants and organizers

A notable shortcoming was a lack of sufficiently articulating and communicating mutual expectations a priori. In the survey, 18.2% of responses mentioned some form of struggling to manage participants’ expectations, but this is likely a low estimate, given that other efforts implied similar issues through the envisioned changes they described for their efforts.

“Participant expectations” included information about what prior knowledge or skills participants should have, guidelines about what participants and practitioner-leaders would provide, and goals for what everyone should gain from participating or leading. Unclear or insufficient discussions of necessary background, participant roles, and scope of efforts were reported as leading to frustration and disappointment. For example, organizers reported that participants without sufficient background information found sessions unapproachable or intimidating. Similarly, when ad hoc efforts tried to foster mentor–mentee relationships, frustration and disappointment often arose on both sides of the relationship when expectations of both parties were not clearly discussed at the start of the relationship.

#### Challenges bridging diverse skill sets and levels

Diversity of attendants was consistently reported as a goal and positive outcome when achieved. However, with such a breadth of skills, the most commonly mentioned shortcoming (40.9%) was difficulty in getting everyone on the same page. Different skill sets and levels made it challenging to present new material at an optimal pace for everyone. Diverse participants also brought diverse expectations for individual events, which could be hard to satisfy, as has previously been noted by Software Carpentries [31].

#### Difficulty cultivating sustained leadership

Despite feeling rewarded by contributing to educational advancement of participants, 22.7% of responses mentioned that organizers reported burnout as a serious consideration. Ad hoc efforts are exciting and meaningful contributions to the data science and institutional communities, but they often go unrewarded or even unacknowledged within traditional academic structures. As a result, organizers struggled to find additional help or people to continue their efforts, which often drove the future of an effort into uncertainty.

#### Difficulty maintaining sustained engagement

Sustaining engagement among participants was another challenge mentioned in nearly a quarter of responses (22.7%). Eliciting initial excitement for data science projects and events was easy, but converting that excitement into regular event attendance, volunteering for presentations, or research output was much more difficult. Due to the extracurricular nature of ad hoc education efforts, there was often insufficient incentive to motivate continued engagement for both practitioner-leaders and participants.

## Discussion

Here, we have considered both multiple-choice and open-ended responses from a survey of organizers of ad hoc education efforts in data science across the MSDSEs. From these, we have generated a taxonomy of ad hoc efforts, have created a codebook for extracting themes from open-ended responses, and have provided a series of lessons learned that emerged from explicit comments from individual organizers and a broader consideration of the responses as a whole. Again, the extent to which these lessons have been experienced by efforts is—if anything—underreported because of the open nature of the survey; the pervasiveness of these (and potentially other) areas of strength and areas for improvement merits further investigation. In this discussion, we build on these reflections of past work to provide concrete suggestions for future ad hoc efforts. We then turn to consider a number of open questions facing ad hoc efforts in data science education that arise naturally from our data and articulate some of the limitations of our work.

### Suggestions for ad hoc education efforts

Despite the successes of past ad hoc education efforts, there remain areas for improvement that can help guide plans for future events. In particular, there is a need for better communication and more conscious planning. Importantly, although the suggestions listed here are informed by the survey of MSDSE effort organizers, these suggestions equally apply to all practitioner-leaders, not just those affiliated with the MSDSE initiative, and some have also been cited as lessons or suggestions in previous work that has looked at individual educational effort formats. These suggestions may be most valuable to practitioner-leaders from institutions with lower levels of institutional support and/or smaller existing data science communities, as these suggestions can help make the most of the available opportunities, time, and resources. Adopting these suggestions can help improve both the quality of individual ad hoc efforts and the quality of ad hoc education—and data science practice—more broadly.

#### Survey participants before and after events

Nearly a third of respondents (31.8%) reported surveying participants either before or after events and noted the utility of that information in shaping their current and future efforts. (Several other respondents noted that they would like to adopt pre- or postevent surveys in the future, and 13.6% noted regretting that they did not have success metrics from surveys.)

Surveys prior to events provided leaders and organizers with essential insights for effectively planning an event. It can help practitioner-leaders set an appropriate pace for tutorials and projects, help organizers manage prospective expectations, and help organizers decide how time should be allocated in longer events (e.g., how to partition time between tutorials and hacking sessions during a hack week) [20]. Participant surveys conducted after an effort are valuable for gathering feedback for improvement and for gathering metrics of success that could then be used to evaluate efforts and bolster support for future instances of the effort [8, 13, 20, 24, 30–32].

#### Communicate goals to manage expectations

Organizers should carefully articulate the goals of the event to practitioner-leaders before the event to identify the minimum knowledge or skills that will be required to participate fully in the event. Articulating and communicating goals and expectations was noted as a challenge in the responses to our survey, consistent with a number of lessons or suggestions for individual formats identified by previous work [20, 24–26, 29, 32]. These goals and requirements should then be shared with participants to improve understanding—and manage expectations—as also noted in related work [32]. If possible, this information should be prominently shared when soliciting participation so that participants can take that information into account when deciding whether to join the event, especially for multiday events like workshops and hackathons.

#### Communicate necessary prior knowledge

Articulating the effort’s goals and target audiences will help organizers to decide how to manage the tradeoff between required participant preparation and the speed and depth of the ad hoc education effort. Additionally, organizers could identify ways that the participants could prepare for the effort, as suggested by 27.3% of respondents in our survey. Organizers should include any essential requirements in the recruitment materials so that participants have a clear understanding of what prior experience (if any) is needed to benefit from the event and so that participants can arrive prepared for the effort. It is important to set expected knowledge at an appropriate level, as setting a high bar of required skills may discourage potential participants with little data science background and thus decrease diversity.

#### Engage representatives to foster diversity

Articulation and communication of event goals are particularly important for efforts that seek to engage diverse audiences. Ad hoc education efforts present a fantastic opportunity to creatively reach audiences that are cross-disciplinary and underrepresented within data science. However, organizers should actively work to reach these audiences as leaders and participants, and effective efforts are unlikely to organically materialize without explicit articulation and dedicated planning. This is reflected by the challenges faced by some respondents in successfully engaging diverse audiences (18.2%) and by the near-majority of efforts reporting that they would make changes to address issues of diversity (45.5%), including one respondent explicitly advocating for diversifying the effort leadership.

Efforts seeking to reach diverse disciplines or demographics should identify clear steps to successfully achieve that goal, as related efforts have also reflected [20]. If an effort intends to target participation by diverse research fields, organizers should reach out to representatives from those areas early in the planning process, either to include the representatives in the organizing process or to request feedback on organizational structure. This is especially important when practitioner-leaders come from more computationally minded fields (e.g., computational biology) and are reaching out to audiences from less traditionally computational fields (e.g., social sciences). Partnering with representatives can provide invaluable insights into engaging and servicing the target audience, including suggestions on material that should be covered, use-case examples to effectively translate and demonstrate skills, or even help advertising within that community.

#### Support development of soft skills

Organizers should also consider how practitioner-leaders will benefit from engaging in the ad hoc effort to help sustain broad engagement of practitioner-leaders. Many soft skills—such as management, public speaking, presenting, communicating, and teaching—are invaluable for any field or career track. Ad hoc education efforts provide wonderful opportunities for practitioner-leaders to practice these skills, but additional support for development would benefit the leaders and potentially improve the incentives for participation. Financial incentives are a possible option, but alternative models of support may be considered, such as providing constructive presentation feedback from the audience, suggestions for developing mentor–mentee relationships [25], or organizers to presenters and building camaraderie among mentors. Organizers could even open a dialogue with potential practitioner-leaders directly to see what might be the most useful benefits to provide.

#### Avoid duplication

Conducting duplicate or significantly overlapping efforts at the same institution is not always the best use of resources. These can generate unnecessary time constraints on organizers and individuals who try to participate in too many overlapping efforts. This may have been of particular concern in our survey, as it targeted the coordinated MSDSEs, but ad hoc efforts can be cross-institutional, a situation in which this concern could be exacerbated. It is also relevant to any large institution where, e.g., multiple departments may rely on ad hoc efforts to teach coding skills. Individuals’ oversubscription to overlapping efforts can exacerbate problems with follow-through and burnout. The coordination of efforts within institutions or cross-institution communities and disciplines remains a difficult but important concern. It is most acutely felt at institutions with relatively lower levels of institutional support or with relatively smaller data science communities.

#### Work towards continuity, reproducibility, and scalability

One possible way to help coordinate education efforts may be by using the tools of scientific reproducibility that have already become a staple for data science (e.g., open code repositories like GitHub and the Open Science Framework). By openly sharing these materials, organizers of new efforts can see what topics have been covered by other efforts and prevent the unnecessary duplication of efforts by reusing existing materials as appropriate [18, 22, 33]. Efforts that have generated a stable repository of education materials reported this to be a major achievement and benefit for future sessions. Some efforts are actively working to address these questions [13], and future work may seek to document the impact and uptake of shared learning materials.

In addition to providing lasting resources for ad hoc effort participants, adopting open science principles may facilitate incorporating particularly relevant and successful ad hoc efforts into formal curricula components. Using such tools may be most impactful by serving as vehicles to replicate and spread expertise to smaller and less well-funded institutions.

### Open questions

The shape of ad hoc efforts will undoubtedly change at the MSDSEs and beyond as data science matures. As such, many questions remain facing ad hoc education efforts for data science. Through survey responses and conversations with other data science educators and researchers, we have identified a few open questions that will likely influence the future of ad hoc efforts at the MSDSEs and beyond by contributing to conversations at the intersection of ad hoc efforts and formal data science curricula. The open questions that follow are meant to engage education-focused members of the entire data science community as they work together to identify a range of solutions that can address a variety of institutional, domain, and individual needs.

#### To what extent will formal educational opportunities that emerge for data science diminish the need for ad hoc education efforts?

Changes in formal curricula are unlikely to entirely eliminate the need for any ad hoc efforts. This is evident from the existence of ad hoc efforts (e.g., informal research projects, summer schools, lecture series) in mature disciplines (e.g., biology, physics) and because some strengths of ad hoc efforts have been much more difficult for formal curricula to achieve (e.g., improving diversity, providing approachable introductions). However, the nature and content of ad hoc efforts will undoubtedly change as formal education efforts in data science grow and as novel formats for curricula are considered across departments [34]. For example, basic programming skills and model interpretation are being increasingly taught in many departments [5, 6, 8–10], degree programs in data science are proliferating [12], and some universities are beginning to require introductions to computer science. Incorporating some of the skills taught in ad hoc efforts into formal curricula will likely change the balance of ad hoc efforts and curricula, potentially lessening the need for some ad hoc efforts.

#### How can we identify the often overlooked institutional infrastructure that already supports ad hoc efforts?

Although many of the respondents did not explicitly address it, the institutional infrastructure within and across the MSDSEs has been an essential element in the successes of ad hoc efforts. As a result, it is important to recognize the invisible infrastructure that makes this possible at institutions: dedicated co-working spaces that are perfect for these events, administrative staff that support logistics and communications, a wealth of knowledge shared freely throughout sibling programs, and funding for scholars across career stages to work collaboratively. These have been key for the success of the ad hoc efforts run across the MSDSEs and are, arguably, the most difficult to reproduce given the financial investment. In order to expand access to ad hoc data science education, we must identify these invisible contributors to success at high-resource institutions and then attempt to identify solutions that can accommodate a range of resource availabilities at other institutions.

#### To what extent should ad hoc efforts facilitate replication at resource-poor institutions?

While ad hoc efforts at individual institutions have provided data science support for some individuals, it is unclear how to scale efforts not only within institutions but also between institutions. Generating material and support for implementing efforts outside of the MSDSEs—especially at institutions with varying resources—is a particularly important area for consideration. Like the previous open question, addressing these disparities in resources and outcomes will take a concerted effort across a range of institutions. Ultimately, creating a variety of different ad hoc data science education effort models may allow lower-resource institutions greater flexibility in identifying models that can work for them. However, answering this question will take additional work and must incorporate more diverse voices: The institutions that we have considered share similar profiles as large research institutions and therefore may not have lessons that generalize to institutions with different profiles.

### Limitations and future directions

This work is a first step in examining the ad hoc data science education landscape, so it has various limitations that provide avenues for future work.

First, our survey targeted only efforts held at the MSDSEs, which are coordinated efforts at institutions of somewhat similar profiles (i.e., large research universities in the United States). Thus, lessons learned might need adaption for efforts at institutions of different profiles with different focuses, resources, and communities. Further work is needed to fully generalize to data science education beyond the MSDSEs. For example, the high level of targeted investment in data science through the mission of the MSDSEs—along with the general level of resources available at the host institutions—present a certain set of ad hoc effort opportunities, and there may be unique pressures, concerns, and opportunities at institutions with different profiles that cannot be readily seen in our survey. Future work should target a broader range of institutions to compare and contrast their needs and experiences.

Second, our work is grounded in a largely open-ended survey of organizers of these events and is limited to their subjective perceptions, which may be biased. We were concerned about potential positive bias in reporting retrospectives, so we designed the survey to try to produce a holistic and balanced view of each event: Out of the 6 open-ended questions asked, only 1 question explicitly asked organizers to describe their successes, while 3 questions were designed to get organizers to think about limitations of their effort. However, organizers may still have unintentionally responded more positively due to their personal involvement in the efforts, as has been established by behavioral research on response bias (e.g., [35]).

Third—and related to the previous limitation—we did not collect data on participants’ subjective experiences or on objective learning outcomes. Some previous work has looked to empirically examine participants’ perceptions and learning outcomes (e.g., [13, 19, 20, 30, 31]), and the present work is intended to complement that work. Future work should attempt to bridge these 2 perspectives quantitatively and qualitatively. Special attention should be paid to whether the organizers’ goals and perceived benefits match participants’ expectations and experiences. These follow-ups are especially important given recent mixed findings on whether short-format trainings—such as boot camps—are [13, 19, 20, 31] or are not [30] effective.

Finally, shortcomings (and successes) are likely underreported because codes were derived from responses to open-ended questions. A more accurate count might come from creating a survey that asks for explicit ratings of closed-form questions. Future work should identify converging ways of evaluating ad hoc efforts by bridging qualitative and quantitative methodologies. One starting point may be to leverage the codebook developed here to inform closed-form surveys or to continue to code open-ended responses.

### Conclusion

While ad hoc efforts (like volunteer research experience and seminar series) have broadly been a staple of academic institutions, ad hoc efforts have played a particularly important role within data science education. The role of ad hoc efforts will likely continue to rapidly evolve with the evolution of data science itself—especially as the field grows to encompass formal courses, degrees, divisions, and departments.

We explored a variety of ways that ad hoc education efforts have attempted to complement formal curricula, along with important considerations that can increase the likelihood that these efforts meet their desired impacts. Additional qualitative and quantitative work is needed, but our discussion of the lessons learned across the MSDSEs will allow future efforts to improve upon past efforts and to benefit a wider audience.

Here, we developed a new codebook that may be used to ground future evaluations of ad hoc efforts. We then used that codebook to extract insights, suggestions, and recommendations that will allow active and future practitioner-leaders from a variety of fields—in computational biology and beyond—to improve their educational outreach. By presenting this synthesis of ad hoc education efforts in data science to practitioner-leaders, we seek to inform conversations about refining these efforts, understanding their place in data science education, and shaping the future of data science education.

## Materials and methods

We sought to compile an understanding of what types of ad hoc efforts have been developed and to extract a series of lessons learned from these responses.

### Efforts surveyed

To find a diverse yet tractable group of ad hoc efforts to survey, we considered the efforts undertaken across the MSDSEs. We sought to include every educational effort held at an MSDSE that did not necessarily provide any course credit and was not required to complete a degree program. In some cases, students could apply for independent study to receive credit for extended (e.g., semester-long) ad hoc efforts, but this was not universally the case.

The MSDSEs were the Center for Data Science at NYU (https://cds.nyu.edu), the Berkeley Institute for Data Science at UC Berkeley (https://bids.berkeley.edu), and the eScience Institute at UW (https://escience.washington.edu). These sibling initiatives were charged with advancing the intersection of domain sciences and data science, making them a prime test case for understanding the state of ad hoc education efforts today.

### Data collected

To learn from the MSDSEs’ ad hoc education efforts, we contacted the organizational leads of the MSDSE environments to inquire about what events they already knew were happening, compiled a preliminary list of efforts held, and contacted organizers of those events with an online survey. We sought to include every educational effort that was not designed to offer course credit or be needed to complete a degree program at one of the universities. Links to the survey were also sent via email to general listservs at each of the 3 MSDSE institutions. These complementary approaches allowed us to target known organizers of known efforts and to solicit responses from a broader range of efforts and individual organizers.

The survey consisted of 2 multiple-choice questions about goals and audiences (see Table 3), questions for logistics, and 6 open-ended questions targeting 4 main areas: (a) the description of the effort, (b) its strengths and weaknesses, (c) lessons learned, and (d) suggestions for future efforts. The exact wording for these open-ended survey questions is provided in Table 4. This survey was designed to get organizers to think critically about their effort and elicit a balanced perspective on each effort in context.

**Table 3:** Multiple-answer survey questions presented to ad hoc education effort practitioner-leaders.

	Survey Question	Options
**1**	What was the goal of the effort?	• Improve coding ability, including ability to use libraries and new tools • Improve knowledge of statistical methods • Exposure to research • Mentoring or career development • Other (open-ended)
**2**	What was the target population of your effort?	• None—no specific target group • Undergraduate students • Graduate students • Postdocs • Diverse academic disciplines • Low coding experience • Diverse demographics (i.e., including individuals from underrepresented groups) • Students interested in academia • Students interested in industry • Other (open-ended)

**Table 4:** Open-ended survey questions presented to ad hoc education effort practitioner-leaders.

	Survey question
1	What was successful about the effort?
2	What was unsuccessful about the effort?
3	What would you change about this effort?
4	What suggestions for future efforts do you have as an outgrowth of this effort?
5	Do you have any other lessons to share?
6	Do you have any thoughts about the future of ad hoc education efforts (e.g., gaps to be filled, suggestions for new formats, efforts that are becoming less useful)?

In addition to quickly incorporating and disseminating emerging methods and tools through focused efforts that deploy quicker than curricula, ad hoc education efforts can meet other needs that have not been fully served by curricula. While many universities are innovating to address data science education (including initiatives at UC Berkeley [http://data.berkeley.edu/], NYU [http://datascience.nyu.edu/], and UW [http://escience.washington.edu/education/]), we identified 4 key areas in which ad hoc education efforts could strive to support community needs: improving coding ability, improving practical knowledge of statistical methods, exposure to research, and mentoring and career development. Similarly, we identified 9 possible audiences that ad hoc efforts might target. Respondents were able to indicate which, if any, of these audiences and goals they had in mind, and they were able to input additional audiences and goals that we did not provide to specify effort intentions.

### Analysis

To extract lessons learned and suggestions for ad hoc efforts, the first and second authors used inductive coding research methods from ethnography and other qualitative research to analyze practitioner-leaders’ open-ended responses through close, iterative contact with the data [27, 28, 36]. These standard methods in qualitative research allow for grounded and inductive insights from open-ended data or a mix of open-ended and structured data (e.g., [37, 38]).

The first and second authors began by reviewing the responses together. The first author then created an initial codebook of relevant themes taken from considering the answers holistically. The first and second authors then worked to refine the codebook together through another round of independent coding while discussing the codebook. The 2 authors retained the codes that both authors individually rated as applying to at least 2 distinct efforts. The first and second authors then coded the analyses together to come to full agreement on all final codes that are discussed here, similar to previous work in this area [39]. The final codebook and resulting codes formed the foundation for the analyses presented here (see Table 5); as noted earlier, we see the the resulting codebook as a product of this research that could be useful for future studies exploring ad hoc efforts [39].

**Table 5:** Codebook used to code open-ended responses from survey respondents and statistics on how many responses mentioned a code. Codes were developed using grounded qualitative methodology [27]. Because the survey relied on open-ended questions, the ratings provided here are likely lower than what organizers would report with specific multiple-choice (e.g., Likert-style scales) or polar (e.g., yes/no, true/false) questions.

Category	Code text	Number (%) of responses
**Suggestions**	Leaders surveyed participants before or after the event (before: to assess expectations, skill level, etc.; after: to assess improvement opportunities, goal attainment, etc.)	7 (31.8%)
**Successes**	Leaders reached or engaged diverse skill levels and/or disciplines; event had accessible format	11 (50%)
**Successes**	Participants learned technical skills (including reproducibility) and/or tools; attendance led to improved research	15 (68.2%)
**Successes**	Participants had nontechnical benefits: learned soft skills (e.g., ethics, communication, presenting opportunity, learning how to learn), got mentored, formed collaborations	9 (40.9%)
**Successes**	Leaders created reusable educational outputs for reproducing effort or similar efforts	3 (13.6%)
**Successes**	Leaders reported a feeling of significant participation: i.e., lots of participation at the time of the event or lower participation over a long period (e.g., community building)	9 (40.9%)
**Shortcomings**	Leaders felt insufficient participation/momentum for the effort (e.g., low engagement from participants, insufficient preparation by leaders, or insufficient momentum/investment in continuing effort)	5 (22.7%)
**Shortcomings**	Leaders had challenge bridging diversity in skill sets, preparation level, experience, etc.; leaders reported feeling it was hard to get everyone on the same page	9 (40.9%)
**Shortcomings**	Leaders struggled to manage expectations of participants	4 (18.2%)
**Shortcomings**	Leaders reported that they didn't gather any/enough metrics to justify success of effort and wish they had	3 (13.6%)
**Shortcomings**	Leaders had insufficient bandwidth to support unsupported effort in the short or long term (e.g., leaders were exhausted)	5 (22.7%)
**Shortcomings**	Leaders reported lack of success in achieving some kind of diversity (e.g., skill sets, demographics, career stages, disciplines)	4 (18.2%)
**Changes**	Leaders reported that they did or would change something format specific (e.g., reorganization, logistics) for a future version of this effort or similar efforts	19 (86.4%)
**Changes**	Leaders did or would like to address issues of diversity in future efforts (e.g., broadening skill levels, addressing imposter syndrome), including by recruiting and selecting to engage more diversity (in participants and leadership)	10 (45.5%)
**Changes**	Leaders reported that they did or could ask future participants to prepare for the effort in some way (e.g., ask for their expectations beforehand, assess levels of preparedness beforehand)	6 (27.3%)

### Ethics statement

This study was approved by the UC Berkeley IRB, and we received written consent from participants, as according to the approved protocol.

## References

[1] Zweben S, Bizot B. 2017 CRA Taulbee Survey. Computing Research News. 2018;30(5):1–47.

[2] Cao L. Data science: a comprehensive overview. ACM Computing Surveys (CSUR). 2017;50(3):43.

[3] Allen G, Benger W, Hutanu A, Jha S, Löffler F, Schnetter E. A practical and comprehensive graduate course preparing students for research involving scientific computing. Procedia Computer Science. 2011;4:1927–1936.

[4] Atwood TK, Bongcam-Rudloff E, Brazas ME, Corpas M, Gaudet P, Lewitter F, et al GOBLET: The global organisation for bioinformatics learning, education and training. PLoS Comput Biol. 2015;11(4):e1004143 10.1371/journal.pcbi.1004143 25856076 PMC4391932

[5] Baumer B. A data science course for undergraduates: Thinking with data. The American Statistician. 2015;69(4):334–342.

[6] Çetinkaya-Rundel M, Rundel C. Infrastructure and tools for teaching computing throughout the statistical curriculum. The American Statistician. 2018;72(1):58–65.

[7] Clark D, Culich A, Hamlin B, Lovett R. BCE: Berkeley's common scientific compute environment for research and education. In: Proceedings of the 13th Python in Science Conference (SciPy 2014); 2014. p. 5–13.

[8] Hill BM, Dailey D, Guy RT, Lewis B, Matsuzaki M, Morgan JT. Democratizing Data Science: The Community Data Science Workshops and Classes In: Big Data Factories. Springer; 2017 p. 115–135.

[9] Jacobs CT, Gorman GJ, Rees HE, Craig LE. Experiences with efficient methodologies for teaching computer programming to geoscientists. Journal of Geoscience Education. 2016;64(3):183–198.

[10] Millman J K, Brett M, Barnowski R, Poline JB. Experiences with efficient methodologies for teaching computer programming to geoscientists. Frontiers in Neuroscience. 2018;12:727 10.3389/fnins.2018.00727 30405329 PMC6204391

[11] The Moore-Sloan Data Science Environments. Creating institutional change in data science; 2018. Available from: http://msdse.org/files/Creating_Institutional_Change.pdf. [cited 2020 Apr 17].

[12] West J, Portenoy J. The data gold rush in higher education. Big Data is Not a Monolith. 2016. Sugimoto C R, Ekbia H R, Mattioli M, "The Data Gold Rush in Higher Education," in Big Data Is Not a Monolith, MITP, 2016. p. 129–139.

[13] Huppenkothen D, Arendt A, Hogg DW, Ram K, VanderPlas JT, Rokem A. Hack weeks as a model for data science education and collaboration. Proceedings of the National Academy of Sciences. 2018;115(36):8872–8877. 10.1073/pnas.1717196115 PMC6130377 30127025

[14] Rokem A, Aragon C, Arendt A, Fiore-Gartland B, Hazelton B, Hellerstein J, et al. Building an urban data science summer program at the University of Washington eScience Institute. In: Bloomberg Data for Good Exchange Conference; 2015.

[15] Wilson G. Software Carpentry: Lessons learned. F1000Research. 2016;3 10.12688/f1000research.7209.1 24715981 PMC3976103

[16] Alnoamany Y, Borghi JA. Towards computational reproducibility: researcher perspectives on the use and sharing of software. PeerJ. 2018;4:e163. 10.7717/peerj-cs.163 PMC7924683 33816816

[17] Wilson G, Aruliah DA, Brown CT, Hong NPC, Davis M, Guy RT, et al Best practices for scientific computing. PLoS Biol. 2014;12(1):e1001745 10.1371/journal.pbio.1001745 24415924 PMC3886731

[18] Holdgraf C, Culich A, Rokem A, Deniz F, Alegro M, Ushizima D. Portable learning environments for hands-on computational instruction: Using container-and cloud-based technology to teach data science. In: Proceedings of the Practice and Experience in Advanced Research Computing 2017 on Sustainability, Success and Impact. ACM; 2017. p. 32.

[19] Stefan MI, Gutlerner JL, Born RT, Springer M. The quantitative methods boot camp: Teaching quantitative thinking and computing skills to graduate students in the life sciences. PLoS Comput Biol. 2015;11(4):e1004208 10.1371/journal.pcbi.1004208 25880064 PMC4399943

[20] Shade A, Dunivin TK, Choi J, Teal TK, Howe AC. Strategies for building computing skills to support microbiome analysis: a five-year perspective from the EDAMAME workshop. bioRxiv. 2019; p. 631267. 10.1128/mSystems.00297-19 PMC6702294 31431509

[21] Via A, Blicher T, Bongcam-Rudloff E, Brazas MD, Brooksbank C, Budd A, et al Best practices in bioinformatics training for life scientists. Briefings in Bioinformatics. 2013;14(5):528–537. 10.1093/bib/bbt043 23803301 PMC3771230

[22] Devenyi GA, Emonet R, Harris RM, Hertweck KL, Irving D, Milligan I, et al Ten simple rules for collaborative lesson development. PLoS Comput Biol. 2018;14(3):e1005963 10.1371/journal.pcbi.1005963 29494585 PMC5832188

[23] Stevens SL, Kuzak M, Martinez C, Moser A, Bleeker P, Galland M. Building a local community of practice in scientific programming for Life Scientists. PLoS Biol. 2018;16(11):e2005561 10.1371/journal.pbio.2005561 30485260 PMC6287879

[24] Sufi S, Nenadic A, Silva R, Duckles B, Simera I, de Beyer JA, et al Ten simple rules for measuring the impact of workshops. PLoS Comput Biol. 2018;14(8):e1006191 10.1371/journal.pcbi.1006191 30161124 PMC6116923

[25] Masters KS, Kreeger PK. Ten simple rules for developing a mentor–mentee expectations document. PLoS Comput Biol. 2017;13(9):e1005709 10.1371/journal.pcbi.1005709 28934208 PMC5608163

[26] Lescak EA, O'Neill KM, Collu GM, Das S. Ten simple rules for providing a meaningful research experience to high school students. PLoS Comput Biol. 2019;15(4):e1006920 10.1371/journal.pcbi.1006920 31022174 PMC6483153

[27] Corbin JM, Strauss A. Grounded theory research: Procedures, canons, and evaluative criteria. Qualitative Sociology. 1990;13(1):3–21.

[28] Chandra Y, Shang L. Inductive coding In: Qualitative research using R: A systematic approach. Springer; 2019 p. 91–106.

[29] Budd A, Dinkel H, Corpas M, Fuller JC, Rubinat L, Devos DP, et al Ten simple rules for organizing an unconference. PLoS Comput Biol. 2015;11(1):e1003905 10.1371/journal.pcbi.1003905 25633715 PMC4310607

[30] Feldon DF, Jeong S, Peugh J, Roksa J, Maahs-Fladung C, Shenoy A, et al Null effects of boot camps and short-format training for PhD students in life sciences. Proceedings of the National Academy of Sciences. 2017;114(37):9854–9858. 10.1073/pnas.1705783114 PMC5604013 28847929

[31] Aranda J. Software carpentry assessment report; 2012. Available from: https://software-carpentry.org/files/bib/aranda-assessment-2012-07.pdf. [cited 2020 Apr 17].

[32] Via A, De Las Rivas J, Attwood TK, Landsman D, Brazas MD, Leunissen JA, et al Ten simple rules for developing a short bioinformatics training course; PLoS Comput Biol. 2011;7(10):e1002245 10.1371/journal.pcbi.1002245 22046119 PMC3203054

[33] Batut B, Hiltemann S, Bagnacani A, Baker D, Bhardwaj V, Blank C, et al Community-driven data analysis training for biology. BioRxiv. 2018; p. 225680. 10.1016/j.cels.2018.05.012 PMC6296361 29953864

[34] Gutlerner JL, Van Vactor D. Catalyzing curriculum evolution in graduate science education. Cell. 2013;153(4):731–736. 10.1016/j.cell.2013.04.027 23663771

[35] Rosenman R, Tennekoon V, Hill LG. Measuring bias in self-reported data. International Journal of Behavioural & Healthcare Research. 2011;2(4):320. 10.1504/IJBHR.2011.043414 PMC4224297 25383095

[36] Thomas DR. A general inductive approach for analyzing qualitative evaluation data. American Journal of Evaluation. 2006;27(2):237–246.

[37] Kross S, Guo PJ. End-user programmers repurposing end-user programming tools to foster diversity in adult end-user programming education. In: 2019 IEEE Symposium on Visual Languages and Human-Centric Computing (VL/HCC). IEEE; 2019. p. 65–74.

[38] Graziotin D, Fagerholm F, Wang X, Abrahamsson P. Consequences of unhappiness while developing software. In: Proceedings of the 2nd International Workshop on Emotion Awareness in Software Engineering. IEEE Press; 2017. p. 42–47.

[39] McDonald N, Schoenebeck S, Forte A. Reliability and inter-rater reliability in qualitative research: Norms and guidelines for CSCW and HCI practice. Proceedings of the ACM on Human-Computer Interaction. 2019;3(CSCW):72.

